# Technical Challenges for CTC Implementation in Breast Cancer

**DOI:** 10.3390/cancers13184619

**Published:** 2021-09-15

**Authors:** Rocío Ramos-Medina, Sara López-Tarruella, María del Monte-Millán, Tatiana Massarrah, Miguel Martín

**Affiliations:** Servicio de Oncología Médica, Instituto de Investigación Sanitaria Gregorio Marañon (IiSGM), Hospital General Universitario Gregorio Marañón, CIBERONC, Universidad Complutense, 28007 Madrid, Spain; rocio.ramos@iisgm.com (R.R.-M.); sara.lopeztarruella@salud.madrid.org (S.L.-T.); maria.delmonte.externo@salud.madrid.org (M.d.M.-M.); tatiana.massarrah@salud.madrid.org (T.M.)

**Keywords:** CTCs, breast cancer, CTC clusters, liquid biopsy, body fluids

## Abstract

**Simple Summary:**

Liquid biopsy appears as a feasible opportunity to complement tissue biopsy within breast cancer research and real-time monitoring of the disease. In this review, we not only summaries cur-rent trends in the study of CTCs as a potential biomarker of response to systemic therapies but also the technical challenges for their implementation in clinical practice, including the role of free circulating DNA and new approaches based on the isolation of CTCs from body fluids. We also highlight the importance of a consensus on the standardization of liquid biopsy methodologies as a fundamental requirement for their implementation in clinical practice.

**Abstract:**

Breast cancer is the most common neoplasm in women worldwide. Tissue biopsy, currently the gold standard to obtain tumor molecular information, is invasive and might be affected by tumor heterogeneity rendering it incapable to portray the complete dynamic picture by the absence of specific genetic changes during the evolution of the disease. In contrast, liquid biopsy can provide unique opportunities for real-time monitoring of disease progression, treatment response and for studying tumor heterogeneity combining the information of DNA that tumors spread in the blood (circulating tumor DNA) with CTCs analysis. In this review, we analyze the technical and biological challenges for isolation and characterization of circulating tumor cells from breast cancer patients. Circulating tumor cell (CTC) enumeration value is included in numerous clinical studies due to the prognostic’s role of these cells. Despite this, there are so many questions pending to answer. How to manage lymphocytes background, how to distinguish the CTCs subtypes or how to work with frozen samples, are some of the issues that will discuss in this review. Based on our experience, we try to address these issues and other technical limitations that should be solved to optimize the standardization of protocols, sample extraction procedures, circulating-tumor material isolation (CTCs vs. ctDNA) and the very diverse methodologies employed, aiming to consolidate the use of CTCs in the clinic. Furthermore, we think that new approaches focusing on isolation CTCs in other body fluids such as cerebrospinal or ascitic fluid are necessary to increase the opportunities of circulating tumor cells in the practice clinic as well as to study the promising role of CTC clusters and their prognostic value in metastatic breast cancer.

## 1. Introduction

Worldwide, female breast cancer (BC) is the most commonly diagnosed cancer (11.7% of all cases), being the leading cause of death, followed by colon cancer in terms of incidence and lung cancer in terms of mortality [1]. Breast cancer is a heterogeneous disease with several molecular subtypes and different histological types, leading to different risk factor profiles as well as different response to treatments and therefore different prognosis. In developed countries, 3 out of 4 cases of breast cancer are diagnosed in postmenopausal women, although about 10% are diagnosed in women under 40 years of age [2]. It should be noted that after 10 years, 30% of breast cancer patients without lymph node metastasis develop metastatic disease, but about 50% of patients with lymph node involvement and local treatment are cured [3]. Some studies suggest that the dissemination of tumor cells could occur in the early stages of disease progression. However, detection in the pre-symptomatic stage is very complicated [4]. Currently, we do not have reliable tools to stage disease progression [5].

The concept of tumor heterogeneity defines the existence at the same time of cellular subpopulations that differ from each other in their genetic and phenotypic characteristics, etc. This heterogeneity is also present between the primary tumor and its metastases. Among the causes that lead to this diversity are genetic and epigenetic factors and mechanisms such as adaptive responses, among others [6]. Techniques such as cytogenetic analysis, chromosomal analysis and microarray-based comparative genomic hybridization (CGH) have been used to demonstrate intra-tumor genetic heterogeneity in breast cancer [6]. Among the characteristics which conditionate the different subtypes, the expression of specific biomarkers, such as estrogen receptor (ER), progesterone receptor (PR) and human epidermal growth factor receptor 2—HER2, as well as different molecular profiles and tumor morphology are the most relevant [7]. Similarly, data suggest the existence of heterogeneity not only spatially (different subpopulations in different regions of the tumor) but also temporally (differences between the primary tumor and its recurrence) [6]. Thus, it should be taken into consideration that the sample obtained in a biopsy does not represent the totality of the composition of the tumor. The tumor is composed of different tumor cells that differ in their properties and drug resistance [7].

CTCs were first described by Thomas Ashworth. More than a hundred years later, many projects have as their main goal the discovery of efficient CTC detection and isolation techniques. The main aim is to apply CTCs as a biomarker of response to systemic therapies [8,9].

**Figure 1:**
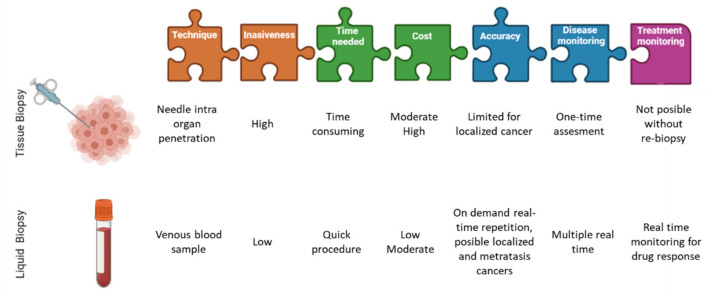
Differences between liquid biopsies and conventional tissue biopsies. Image partially created with BioRender.com and modified from Eslami-S et al. [14].

For this key reason, circulating tumor cells (CTCs) in peripheral blood provide the most objective way to assess intra tumor heterogeneity, facilitating the determination of single cell genotypes, cancer cell origin, differences among the CTCs and the solid tumor as well as those mutations which could address to the potential drug resistances (Figure 1). Thus, the CTCs detection together with optimized analyses methods are very helpful in the clinic for determining intra tumor heterogeneity [7].

Liquid biopsy provides a non-invasive and real-time source of tumor information compared to tissue biopsy. Despite this, this last one remains as the standard for pathological diagnosis because of the value of the information it provides and the need to evaluate biomarkers such as ER or other proteins. However, for metastatic diagnosis, liquid biopsy can play a key role in situations where it is necessary to identify possible targets for targeted therapy [10]. Blood samples are the most common materials for analysis, which contain cell-free DNA (cfDNA); cell-free tumor DNA (ctDNA); vesicles (such as exosomes); tumor-educated blood platelets (TEPs) and circulating tumor cells (CTCs).

Extracellular vesicles appear as a potential source of tumoral biomarkers since they are in fluids circulation and their isolation and analysis seems to be feasible to be implemented in early cancer detection. Both exosomes and other vesicles have been studied by their relevant role in cell signaling and tumoral microenvironment. They have been widely discussed in recent literature highlighting their value to determine the tissue of origin, to monitor response and resistances to treatments and adapt therapy to the specific needs of an individual patient, or to detect minimal residual disease [11,12]. However, validation studies with well-established end-points must be necessary to demonstrate clinical utility and, to introduce them into clinical practice [11].

Other body fluids such as cerebrospinal fluid (CSF); saliva; pleural effusions; urine and stool have shown captivity for diagnoses [13].

The challenges facing CTCs fall into three main blocks: isolation, detection and analysis. CTCs are released from tumors and enter the bloodstream. Once in circulation, their half-life is between 1–2.5 h, which is due, among other things, to attacks by the immune system. Nevertheless, a small number of CTCs survive and give rise to distant metastatic disease [15]. CTCs can circulate in the bloodstream individually or in clusters. CTC clusters consist of primary tumor cells that are held together by plakoglobin-dependent intercellular adhesion. Their presence in the bloodstream is rare, but they have a greater capacity to develop distant metastases than individual CTCs [16,17,18]. Of the total number of CTCs present in the blood, about 3–4% are clusters and in the case of patients with metastatic cancer, about 50% have at least one cluster [19]. However, rarity and heterogeneity of CTCs make them extremely difficult to be detected and utilized as a biomarker [17].

TEPs are now considered to be local and systemic responders to the presence of cancer. They exert their effect both on tumor cells present in the tumor and on circulating tumor cells. Their mechanism of action is divided into several phases, first platelets generate an appropriate environment for neovascularization by providing the tumor with different proangiogenic factors such as VEGF, PDGF and FGF. Subsequently, platelets can induce epithelial-mesenchymal change in tumor cells by direct physical interaction and release of TGF molecules and have also been shown to reduce apoptosis and programmed cell death of local tumor cells. Once in the bloodstream, platelets are likely to protect circulating tumor cells from the immune response, favoring the metastatic process. Thus platelets are a fundamental component of the tumor microenvironment as they participate in tumor initiation, progression and response to therapy [20].

In the past few years, several clinical studies in breast and prostate cancer patients have highlighted the prognostic value of the detection and characterization of CTCs, suggesting they could even be categorized as follow-up markers and guide personalized treatment decisions. The idea of replacing tumor tissue biopsies to obtain diagnostically and therapeutically relevant information makes CTCs an essential contribution to non-invasive “real-time liquid biopsies” [21]. The next step in translating CTCs as liquid biopsy into clinical practice is to demonstrate the utility of these biomarkers. Observational clinical trials have already shown that CTCs and ctDNA are clinically relevant for different types of cancer. However, interventional clinical trials are needed to demonstrate their utility and to include liquid biopsies in clinical guidelines. Many clinical trials already include these determinations as part of specimen testing. However, the extent to which liquid biopsy could eventually replace tissue biopsies remains to be clarified. We know that for diagnosing primary tumors or staging metastatic lesions in tissues that are difficult to sample, liquid biopsy could be a reliable alternative [22]. Focusing on breast cancer, the biopsy of metastatic tissue is sometimes not clinically possible due to its location (lung) complicating the correctly determination of the molecular profile of a tumor by the limitation of taking biopsies from different tumor sites [22].

## 2. Clinical Trials with CTC-Based Treatment Decisions

Regarding breast cancer, the isolation and quantification of CTCs in early or locally advanced or metastatic breast cancer has shown independent prognostic value in several clinical trials and meta-analyses [23]. Even though, due to their limited number, the clinical assessment of CTCs in the early stages of the disease is limited. If we focus on non-metastatic breast cancer, we find less than 1CTC/10 mL of blood, with five or more CTCs (1–5.9%) being considered a rare event. At diagnosis, we can detect CTCs in about 20% to 25% of patients with localized non-metastatic breast cancer using a lower threshold (≥1 CTC per 7.5 mL of blood) than in metastatic breast cancer (≥5 CTCs per 7.5 mL of blood) [24]. Neoadjuvant and adjuvant studies in early breast cancer have shown a moderate association of CTC detection with positive lymph nodes, but not with any of the other classical prognostic factors, nor with tumor subtype. The aim of the IMENEO study, a meta-analysis conducted in more than 2000 non-metastatic BC patients from 16 centers treated with neoadjuvant chemotherapy (NCT), was to evaluate the clinical validity of CTC detection as a prognostic marker. The results showed a statistical significance of CTC count increased with the number of CTCs, from none to one cell, with an HR of 6.25 (95% with confidence interval [CI] = 4.34 to 9.09) for five or more cells, reinforcing the concept of CTC count as a quantitative marker [24]. Some studies have demonstrated an increased risk of recurrence in patients with persistent CTCs for two years and even five years after (neo)adjuvant chemotherapy (SUCCESS-A study and ECOG-ACRIN study E5103).

In a secondary analysis of the E5103 trial, the authors addressed the interesting role of CTCs enumeration, 5 years after diagnosis, in the prediction of high-risk hormone receptor-positive HER2-negative BC late recurrences. CTC-positive patients recurrence rates per person-year of follow up where 21.4% vs. 2.0% in CTC-negative, and the single positive CTC detection retained its independent prognostic value adjusted for multiple clinical covariates.

Specifically, in the SUCCESS-A study, the detection of CTCs after two years of follow-up was associated with a 3.9-fold increased risk of death and a 2.3-fold increased risk of recurrence. In the case of a 5-year follow-up with known CTC value, the study showed that CTC positivity was found in 7.8% and was associated with a 6-fold increase in recurrence [25,26].

One of the most relevant studies carried out in patients with metastatic breast cancer is the one conducted by Cristofanilli et al. in 2004, in which they demonstrated that the detection of CTCs has prognostic value during the disease. According to their data, CTCs were detected in approximately 60% of patients, with a CTC count of ≥5 cells per 7.5 mL of blood associated with significantly worse progression-free survival (PFS) and OS. These results supported the clinical validity of CTCs, opening up the possibility for several research projects investigating to include the assessment of CTCs in the response evaluation to therapy [27]. A recent extensive review of data from 2436 metastatic breast cancer patients from 17 European centers and the MD Anderson Cancer Center in the U.S also deemed this cut-off appropriate. Moreover, Cristofanilli et al. in 2019 reported that patients, who were below the cut-off and, therefore, stratified as stage IV indolent had a significantly longer overall survival of 37.1 months compared to stage IV aggressive (≥5 cells per 7.5 mL of blood) with 15.4 months. Thus, CTC enumeration may serve as important tool for staging of advanced disease and for disease stratification [28]. The S0500 study (NCT00382018) was the first phase III clinical trial to address the clinical utility of CTCs. The results did not show a significant improvement in survival by an early change in treatment regimen (median progression-free survival (PFS) 4.6 vs. 3.5 months, HR = 0.92, 95%CI = 0.64–1.32; median overall survival (OS) 1.5 vs. 10.7 months, HR = 1.00, 95%CI = 0.69–1.47). However, if the prognostic impact of CTCs could be confirmed the median OS reached 35 months in patients with a low pre-treatment CTC count, 23 months in patients with a high pre-treatment CTC count but a low CTC count at follow-up, and 13 months in patients with a persistently high CTC count. The study findings highlighted that early switching to another standard chemotherapy regimen was not effective in terms of increased survival, in those patients with persistently increased CTCs after a cycle of first-line chemotherapy. Therefore, persistent CTCs could represent a population of chemoresistant tumor cells that again require an alternative therapeutic approach [29]. In Table 1, we showed the current clinical breast cancer trials that involve interventions based on CTC number or phenotype.

It should be noted that knowledge about the functional properties of CTCs remains limited even though relevant advances have been made in the molecular detection and characterization of CTCs down to the single-cell level [21]. Today we face many biological challenges: (a) The sample size, 1CTC/109 cells per 7 mL of blood; (b) The presence of cytokeratin positive cells in the blood of “healthy” individuals; and (c) The heterogeneity within the population of CTCs [31]. Furthermore, technological challenges also remain to be solved as well as the need for standardized procedures for sample processing in the pre-analytical phase, or the establishment of reproducible workflows focused on molecular analysis of liquid biopsy samples [32].

In this short review, we analyze the technical and biological challenges for isolation and characterization of circulating tumor cells from breast cancer patients.

## 3. Technical and Biological Challenges for Isolation and Characterization CTCs

### 3.1. Pre-Analytical Blood Sample Handling

For liquid biopsy to be incorporated into routine clinical practice, consensus needs to be reached on the handling of blood samples before analysis and the technologies used to extract cfDNA and isolate CTCs, or the optimal workflows for their molecular analysis [32,33].

To achieve a standardization, the current new Horizon 2020 SPIDA4P consortium project (2017–2020) is working as a coordination and support action to broaden the portfolio of standards for preanalytical workflows. The aim is to finally generate and implement a comprehensive portfolio of 22 pan-European pre-analytical CEN/ Technical Specifications and ISO/International Standards, addressing important pre-analytical workflows as applied to personalized medicine (www.spidia.eu) (accessed on 15 September 2021) [34,35].

CTCs provide great versatility for multi-omic analysis, not to mention their potential to guide patient therapy. However, the handling and collection of the blood sample before processing is a critical process. As we know, CTCs are rare (typically 1–10 CTCs per 106–108 leukocytes), heterogeneous and expensive to enumerate and isolate. Therefore, blood samples must be processed in situ within hours of collection to preserve the viability of CTCs. This makes the transport of live CTCs in blood samples between centers often not possible due to the high senescence of CTCs. To overcome this impediment, blood is usually placed in blood collection tubes with preservatives, which allows storage for up to 3 days. However, it should be noted that this process also entails a loss of viable cells, which reduces the number of possible downstream applications [33].

Since CTCs are generally very rare it is also important to consider the volume of blood sampled to unify protocols and the implications this has for effective CTCs identification and isolation [36].

### 3.2. Genotype and Phenotype of CTCs in Breast Cancer (BC)

In addition to enumeration, new techniques, which enable to efficiently isolate CTCs from patients’ blood, provide the possibility of detailed molecular analysis, expression profiling and phenotype screening [24].

#### 3.2.1. CTC and Epithelial-Mesenchymal Transition

**Figure 2:**
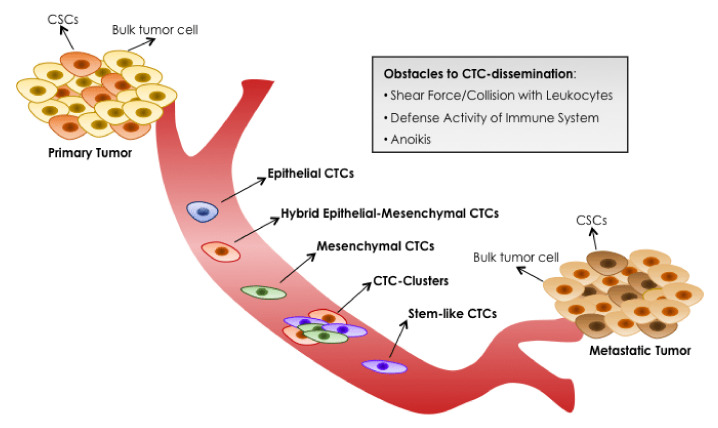
Blood dissemination of different CTCs-subtypes. Primary tumors can shed several CTCs, but only a minim percentage can survive in the bloodstream and only a minority is able to initiate metastatic tumor growth in a distant organ. Mirroring complex heterogeneity of primary tumor cells, phenotypically distinct CTCs-subtypes can co-exist in the circulation. Image created with BioRender.com and modified from Margherita Correnti and Chiara Raggi [40].

Epithelial-mesenchymal transition (EMT) involves the reprogramming of epithelial cells acquiring cellular characteristics representative of mesenchymal cells such as motility, invasion and absence of cell polarity. Different studies have investigated the presence of EMT characteristics in breast cancer CTCs (Figure 2) [37,38]. The genetic changes occurring during EMT lead to increased tumor cell motility and an invasive phenotype. Primarily the changes center on a loss of E-cadherin expression and subsequent translocation of β-catenin from the cell membrane to the nucleus, increased expression of vimentin, production of matrix metalloproteinase enzymes and up-regulation of various EMT-inducing transcription factors, such as Twist, Snail and Slug [39]. The presence of epithelial and mesenchymal subtypes is dynamic during therapy, with a higher presence of mesenchymal CTCs observed at the time of tumor progression [37]. The study by Aktas et al. in metastatic breast cancer patients showed that at least one of the three EMT markers (Akt2, PI3K and Twist1) was expressed in 62% of patients with CTCs. The presence of CTCs with a mesenchymal phenotype increased the likelihood of not responding to palliative chemotherapy, antibodies or hormonal therapy [39].

The role of EMT in the process of metastasis is currently being questioned. Studies have suggested that EMT may not be essential for metastasis in tumors of epithelial origin. In vivo studies performed to confirm the role of EMT in the development of lung metastases from a primary breast cancer tumor indicated that the source of these lesions was not associated with cells derived from the EMT process. These results challenge the original EMT/MET hypothesis [24].

#### 3.2.2. Hybrid Epithelial-Mesenchymal (E/M) Phenotype

Currently, the phenomenon of EMT in oncology is considered more as a succession of phenotypes between mesenchymal and epithelial than as a directional and complete process. Therefore, in the context of tumor cells, it would be more appropriately named as epithelial-mesenchymal plasticity (EMP). Similarly, cells that express both epithelial and mesenchymal markers are identified as epithelial-mesenchymal hybrids (E/M hybrids) [24].

Several studies show that the E/M hybrid phenotype may be the most predictive, being the co-expression of E and M markers in the same CTC associated with cancer progression and lower PFS [24,41,42]. In this regard, the study by Papadaki et al. confirmed that E/M population was increased after chemotherapy in those patients with metastatic breast cancer and without response to treatment consensus. The E/M subpopulation was an independent predictive factor of reduced PFS, and in HER2-negative patients, it was also predictive of decreased OS [43].

The similarity between CTCs of hybrid phenotype and stem cells and the reversible modulation capacity of the latter, makes the functional characterization of these processes in CTCs essential and helpful to clarify the mechanisms of tumor cell dissemination. Therefore, the development of new technologies that improve the sensitivity and efficiency of CTC and CTC cluster detection will facilitate all these studies [39].

#### 3.2.3. ER/PR and HER2 Characterization of CTCs

It is globally accepted that breast cancer is a heterogeneous disease where different molecular and pathological subtypes can be distinguished. Within systemic disease, the study of ER/PR and HER2 expression in CTC has acquired great relevance because of the potential of liquid biopsy to provide therapeutically relevant biomarkers [44].

There are comparative studies between ER/PR/HER2 expression levels on CTCs and primary and metastatic tumors where some discrepancies have been observed. Results suggest that CTCs are more representative of metastatic tumors. In some cases, ER+/PR+ primary breast tumors disseminated CTCs that were ER-/PR-, which could have important implications for treatment decisions for adjuvant and endocrine therapy, such as tamoxifen. On the other hand, concerning HER2 expression levels, there are data demonstrating differences between the level of HER2 expression in ductal breast tumors and CTCs, thus confirming the difference in expression profile between CTCs and primary tumors [4].

To evaluate the rate of conversion of receptor status (HER2, ER and PR) between the primary tumor and the recurrent lesion in patients with advanced breast cancer, the GEICAM 2009-03 ConvertHER study reported that HER2 expression is the most stable while the progesterone receptor showed the highest rate of conversion. Concerning interlaboratory variability, a robust agreement was observed in HER2 expression between local and central laboratories, as well as in ER expression in metastasis. The expression of PR at any site and that of ER at the primary site showed a lower agreement [45].

It is accepted that in almost half of patients with ER-positive primary tumors, ER expression is absent in metastatic lesions. On the other hand, about one-third of HER2/neu-negative primary tumors have HER2 amplification in metastasis. Comparing HER2 amplification expression between the primary tumor and CTCs in non-metastatic breast cancer patients, the discordance can be as high as 49%.

According to the findings of the GEICAM 2009-03 ConvertHER study, although the intrinsic subtype is predominantly maintained during metastatic progression, luminal/HER2-negative tumors acquire a luminal B or HER2-E profile during the metastatic process, highlighting the tumor evolution and the acquisition of oestrogen independence along the disease progress [46]. These discrepancies are mainly due to the lack of consensus on the methodology used and the heterogeneity of the CTCs [5]. However, in case of ER/PR status, comparative analyses revealed a wide range of agreement (40–70%) between matched CTCs versus primary tumors [44].

To date, a consensus has not yet been reached to establish the methodology to assess biomarker status in CTCs and to standardize the criteria for positivity. Until now, these are rational approaches based on the utility of CTCs for the identification of ER and HER2 patients and a clinical validation of the therapeutic prediction of these approaches is needed [44,47].

### 3.3. Cryopreservation and CTCs Isolation

The possibility of developing retrospective studies with tumor samples is the core of clinical research. So far, given the limited storage period of commercial blood collection tubes, the blood sample for the study of CTCs has to be processed on the same day or a few days later. For this reason, cryopreservation offers new scenarios for laboratories, allowing batch processing of blood samples and collection of samples over time for future analysis [48,49]. Additional advantages include performing the assay in batches in a centralized laboratory to minimize interlaboratory variations and assaying longitudinal collections together to minimize inter assay variations [50]. However, a reproducible cryopreservation protocol needs to be established and agreed upon by the research community to confirm that the cryopreserved CTCs are the same as those found in the fresh sample, providing prognostic information about the disease outcomes [51]. The results of the study by Lu et al. show that liquid biomarker determination from frozen samples is feasible and representative of the prospectively collected sample. Morphology, genetic alterations and biomarker localization are preserved compared to fresh samples [48]. One of the main drawbacks of cryopreservation is the possible loss of CTCs due to the freezing, storage and thawing process. In this regard, the study by Nejlund et al. [49], shows that there is no significant loss of CTC in cryopreserved samples compared to their fresh sample. [49]. On the other hand, Friedlander et al. [52] reported that in the case of prostate cancer patients, the process of cryopreservation of PBMCs did not influence cell recovery but, they also observed that the loss of CTCs associated with this process is somewhat more predominant in samples with many CTCs (>50). In Brungs et al. study [51] a tendency to cell clumping was observed in samples with a high number of CTCs despite the presence of benzonase. The cause of this phenomenon is the release of viscous DNA from cell lysis during the thawing process, which results in aggregates, preventing an accurate CTC count so, there is still an unmet need to optimize this process.

So, CTCs cryopreserved at −20 °C or −80 °C can provide a useful way to store the samples in order to reduce the batches or interlaboratory variations and, although a small loss of CTCs after cryopreservation may occur, no statistical differences seem to be reported. Regarding the lymphocyte background, other pending issue to be solved since the isolation of CTCs minimizing the lymphocyte background still lacks standardized protocols among laboratories. At this moment, we would suggest working with isolate single-cell CTCs to assure there is no lymphocyte background.

## 4. New Approaches

### 4.1. Optimize the Standardization of Protocols

Liquid biopsy is a non-invasive methodology, which serves to obtain key tumor information, and it is rapidly transforming the cancer patient’s clinical management. The assessment of the tumor circulating components in the early and advanced setting is very well documented [53]. However, more efficient CTC detection and enrichment technology are needed, including validation of CTCs before they can be incorporated into oncology laboratories. In recent years, the number of clinical trials aimed at assessing the value of CTCs as biomarkers in the treatment of cancer patients has been increasing. That is due to the high demand for minimally invasive diagnostics and an evidence-based medicine approach to treatment selection and monitoring [54]. Despite the numerous platforms developed for CTC analysis, the translation into the clinical practice is still limited [55].

The wide variety of CTC separation technologies and products makes the evaluation and comparison of techniques with each other to establish a standard technique for their study a very tedious task [56]. For future use and approval by health authorities, it is strictly necessary to obtain clinical data to support the standardization of CTC technologies [56]. The importance of reproducibility in CTC detection processes should also not be forgotten, where very few techniques have demonstrated intra- and inter-laboratory reproducibility [37].

In this regard, public-private partnerships have been launched to achieve the standardization of methods and technologies for the analysis of circulating nucleic acids and rare cells. One example is the Innovative Medicines Initiative (IMI) CANCER-ID program (www.cancer-id.eu) (accessed on 15 September 2021). So far, this consortium has evaluated different technologies for the analysis of blood biomarkers and has developed a set of guidelines for the proper conduct of technology comparison studies. These criteria include best practice recommendations for sample collection, protocols for sample storage and shipping to enable biobanking, as well as comparative data on different methods for molecular analysis of CTCs, ctDNA and miRNAs [32].

In summary, although the most standardized method for CTCs isolation is based on immunomagnetic and fluorescence imaging technology other technologies based on microfluidic technologies are breaking into the liquid biopsy scenario [57,58,59,60] there is a clear need for greater specificity, standardization and transparency in the reports to incorporate the detection of CTCs as another tool in clinical practice focused on individualised therapy [61]. Concerning standardisation, this should be implemented in all steps of the process, sample extraction procedures, isolation of ciculating tumor material, etc., so that the use of the different circulating molecules can be extrapolated to the “real world” in clinics. Similarly, the method should be more ambitious with a multi-omic approaches to demonstrate the great clinical utility of liquid biopsy in cancer patients [53].

### 4.2. Isolation of CTCs in Other Body Fluids

There are many other body fluids that can provide a source of tumor-derived molecular information, increasing the value of liquid biopsy as a relevant tool in clinical diagnosis to be explored.

**Figure 3:**
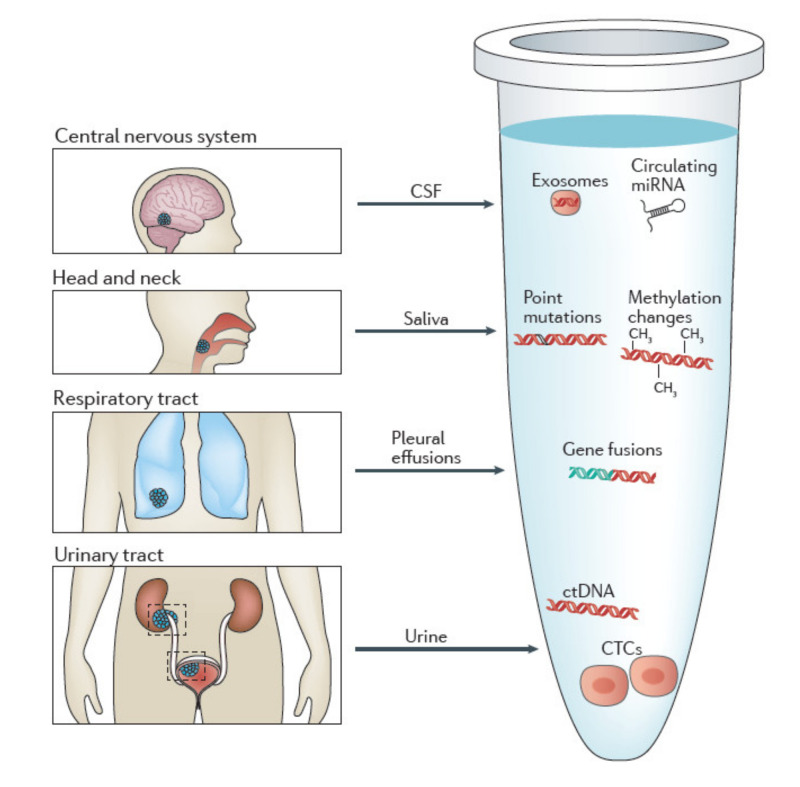
Body fluids as a source of tumor-derives molecular information. Schematic representation of different body fluids (other than blood) that can contain tumor-derived molecular information. Specifically cerebrospinal fluid (CSF), pleural effusions, saliva, urine. Image created with BioRender.com, and modified from Siravegna et al. [74].

The term liquid biopsy is therefore used in contraposition to the traditional surgical tumor biopsy and represents the analysis of cancer biomarkers in tumor-derived material [55]. Even though the “standard” concept of liquid biopsy comprises the sampling of blood, almost all body fluids are suitable for liquid biopsy. Common bodily fluids include urine, saliva, sputum, stool, bile, cerebrospinal fluids (CSF) and pleural effusions (Figure 3) [62].

However, there are hardly any studies where isolation of CTCs from other body fluids is performed; most of the investigations are aimed at isolation of ctDNA. Some of the studied body fluids are listed below.

- Urine

One of the major advantages of using urine is its non-invasive nature of collection compared to tissue or blood, especially in patients requiring repeated sampling to monitor cancer progression and/or therapeutic effects [63].

Currently, there is one clinical trial evaluating urine as a source for liquid biopsy. NCT04432909 is a prospective multicenter, single-blinded study to evaluate the utility of UroCAD for urothelial carcinoma diagnosis and follow-up in 500 participants.

During the past decades, many researchers have performed studies to confirm urine ctDNA as a powerful biomarker not only in urological cancers but also in NSCLC [64], colorectal [65], gastric tumors [66] and breast cancer [67].

Concerning extracellular vesicles (EVs), these cellular elements have become of increasing interest as a potential source of biomarkers due to their role in intercellular communication and the affinity of their molecular content with that of the releasing cells [68].

Several studies have characterized the proteomic profile of urinary EV from urological cancer patients and identified potential biomarkers for bladder cancer and prostate. One of the many studies performed identified urinary EV derived alpha 1-antitrypsin and histone H2B1K as diagnostic and prognostic biomarkers for bladder cancer. When the results were verifying by immunohistochemistry, this confirms significantly higher expression of these markers in bladder cancer tissues than in normal tissues [69].

- Saliva

Depending on the type of cancer different biomarkers can be isolated from saliva, being ctDNA from this source the best biomarker for head and neck squamous cell carcinoma, while miRNAs alone or in association with EVs play a relevant role in the early detection of potentially malignant oral cancers [62].

In 2005, Streckfus, C. et al. showed that C-erb-b2 a breast cancer marker was produced by the salivary glands and detected in the saliva of breast cancer patients [70].

In vitro studies with breast cancer samples have shown that exosomes interact with salivary glands by altering the transcriptome and proteome composition of salivary gland-derived macrovesicles [71].

The results derived from the study by Zhang et al. reveal that transcriptomic and proteomic signatures in saliva can serve as biomarkers for the non-invasive detection of breast cancer. These biomarkers have high specificity and sensitivity, which provide a prediction model validation study for next clinical validation [72].

- CSF

In the case of cancers restricted to the central nervous system (CNS), there is a shortage of circulating biomarkers (ctDNA). For this reason, CSF, due to its direct contact with the CNS, is a relevant source of biomarkers. Studies to date indicate that CSF-derived ctDNA represents the genetic alterations of brain tumors better than plasma ctDNA [62].

In the case of HER2-positive metastatic breast cancer, the CSF DNA has been reported to provide value and additional information to radiological probes.

It has been described in a study comparing CSF and plasma cDNA molecular profiles, a HER2 positive breast cancer patient with leptomeningeal and extracranial metastases, that the clinical and radiological response of each were different to T-DM1 treatment. The CSF cDNA revealed enrichment of ERBB2 amplification, MYC amplification and mutations of the driver genes PIK3CA and TP53, justifying progression in the CNS, while the decrease reflected a partial clinical response in the extracranial compartment [73].

So, ctDNA has two main diagnostic applications in patients with leptomeningeal metastases: (1) completion of the diagnostic profile in patients with negative titration and (2) identification of actionable genomic alterations with the potential to define optimal targeted therapy [73].

- Other bodily fluids

In the case of colorectal cancer (CRC), DNA derived from stool is a powerful diagnostic tool. In lung cancer, sputum protein content and DNA from pleural effusions are highly relevant biomarkers for the diagnosis of lung cancer and malignant pleural mesothelioma [62].

The findings of the case published by Husain et al. in 2017 in a patient with metastatic breast cancer with cytologically active ascites. They highlight the usefulness of cfDNA present in ascitic fluid as a source of additional information that is not detected in free tumor cells and plasma [64].

### 4.3. Promising Role of CTC Clusters

As we have shown in the previous sections of this review, circulating tumor cells are disseminated by the primary tumor and metastases into the bloodstream. We know that different subpopulations exist and that they are responsible for the origin of metastases. It is also accepted that CTCs can be found in the blood as single cells or in clusters [16]. There are two theories about the origin of CTC clusters: (1) the clusters may derive directly from the primary tumor in a process where tumor cells cooperate and migrate collectively; and (2) the CTC cluster arises in the bloodstream by aggregation of individual CTCs [19]. A third theory has now been proposed, called “cell-jamming”. According to this theory, higher extracellular matrix (ECM) density may promote cell clustering and thus facilitate the formation of CTC clusters. Thus, it has been observed that mesenchymal tumor cells show a tendency for collective invasion under conditions of high ECM density [19]. The number of CTC clusters are less than single CTCs, but metastasis is 23–50 times more potent than single CTCs [13]. Studies on the molecular profile of CTC clusters have shown a high expression of the desmosomal binding protein plakoglobin, which may be due to its function in maintaining strong cell-cell contacts in tumoral processes and metastasis [24]. Several studies have also shown the importance of keratin 14+ in the mechanism of cluster metastasis. Keratin 14+ may regulate cell-cell adhesion, cell-matrix adhesion and immune evasion. Moreover, transcription factor binding sites for stem and proliferation, including OCT4, NANOG, SOX2 and SIN3A are exactly hypomethylated in CTC clusters [13]. In contrast, transcripts encoding classical CTC markers such as keratins, mucin 1 (MUC1), EpCAM and E-cadherin are under expressed in CTC clusters [75]. Clusters of CTCs are composed not only of tumor cells but also of cancer-associated fibroblasts, white blood cells and platelets [24,75]. To ensure the viability of tumor cells in the bloodstream, cooperation is necessary. In fact, neutrophils have been shown to increase the metastatic potential of tumor cells by overexpressing the cell cycle and DNA replication genes [75]. To date, clusters of CTCs have been observed in patients predominantly during advanced disease stages (i.e., with established distant metastases). The last study published by Aceto’s laboratory [76] evidences the presence of CTC clusters in early breast cancer, suggesting their possible involvement in early metastatic spread and disease progression.

Finally, Krol et al. have highlighted two implications of identifying CTC clusters in early breast cancer: (1) CTC clusters are a general event of breast cancer biology regardless of disease stage; and (2) The presence of CTC clusters in early breast cancer could be considered as an additional and substantial risk factor for disease progression. However, further clinical trials are needed to confirm the latter point [77].

Summarizing the results of the literature reviewed, it could be said that the CTCs clusters due to their intrinsic heterogeneity provide both spatial and temporal information with a prognostic value, being associated to shorter PFS and OS. Their identification can be solved easily by staining [77] or by separation based on size [78]. Their promising role could be due by their biological activity as a more consistent tool to reproduce primary tumor as compared to tissue biopsy or single CTC providing a possible therapeutic target [79].

## 5. Conclusions

The modern procedure of liquid biopsy has been a breakthrough in clinical oncology and has an extraordinary potential as a tool for BC screening and diagnosis, to estimate prognosis, and both to select targeted-treatments and monitor response. The different layers of information provided by liquid biopsy (CTCs, ctDNA, exosomes) in patients with cancer would help us to better understand tumor biology and evolution. Furthermore, hopefully contribute to advance from prognosis to prediction, this is the real challenge in the era of the so-called precision oncology, being able to identify biomarkers that guide us through the challenging process of deciding which is the most appropriate treatment in each particular moment of each patient’s BC evolution. In this respect, CTCs provide the opportunity for real-time monitoring of disease recurrence, progression and treatment response evaluation. Efforts are undertaken beyond enumeration towards a full characterization of CTCs, and even CTC-clusters, that besides helping to reveal the keys of clonal evolution of a particular tumor, could help clinicians to focus on those dominant clones, scarce living fractions of the tumor, that can better guide treatment strategies as drivers of the metastasis process. Furthermore, the optimization of ex vivo cultures of CTCs could be a valuable model for drug sensitivity testing [80].

According to the available data, it is expected that CTC techniques using whole blood will become a standard procedure. Minimal or no sample preparation is required; therefore, tumor cell damage is minimized, resulting in more reliable CTC analysis of these somewhat limited and precious fraction in the whole blood sample. Technical limitations are in-process to be improved by recent technologies, but a protocol standardization is needed to reach the full potential of this biomarker.

More than likely, even if all the current technological drawbacks are overcome and liquid biopsy reaches the necessary levels of standardization, tissue biopsies will be required today and, in the future, even though they may be invasive. The characterization of the tumor and its microenvironment is key to analyze valuable validated biomarkers (i.e., ER, PR, HER2 receptors in the tumor, or TILs in the microenvironment). However, liquid biopsies hold the promise to add valuable dynamic information and capture a wider landscape of the complex tumor/host interaction that still need to be fully validated. Moreover, CTCs gain special interest in particularly delicate clinical scenarios such as multiorgan metastasis that cannot all biopsied at a time, dissociated tumor responses to given treatments at different metastatic locations, or lesions with complicated access or complicated processing protocols (i.e., bone metastasis). Therefore, in the future, both types of biopsies (liquid and tissue) will act as complementary, providing information about the tumor from different approaches. In this review we ponder some questions that highlight technical challenges still unsolved and necessary for a real CTC-analysis validation before its implementation in the clinical practice.

## Figures and Tables

**Table 1:** Clinical trials with CTC-based treatment decisions. Table modified from Schochter F. et al. [30].

Trail	Condition	Intervention	Primary End Points
Treat-CTC NCT01548677 (phase II)	HER2-neg EBC with CTCs after CT	Trastuzumab iv 6 cycles vs. observation	CTC detection rate at week 18
SWOG S0500 NCT00382018 (phase III)	CT-resistant, CTC-posMBC	Early switch in therapy vs. treatment until progression	OS, PFS
CirCe01, NCT01349842 (phase III)	CT-resistant, CTC-posMBC	Early switch in therapy vs. treatment until progression	OS
STIC-CTC, NCT01710605 (phase III)	HR-pos and HER2-neg MBC	Decision CT or ER by clinical choice vs. CTC count	PFS, economic value
DETECT III NCT01619111 (phase III)	HER2-neg MBC and HER2-pos CTCs	Standard treatment vs. Standard treatment + lapatinib	CTC clearance
DETECT IV NCT02035813 (phase II)	HER2-neg MBC and HER2-neg CTCs	A: ET + ribociclib or everolimus B: eribulin	A: CTC clearance B: PFS
DETECT V NCT02344472 (phase III)	HR-pos, HER2-post MBC	Trastuzumab/pertuzumab + CT or ET with ribociclib	Tolerability, safety and quality of life

CT = chemotherapy, CTC = circulating tumor cells, ET = endocrine treatment, HER2 = human epidermal growth factor receptor 2, HR = hormone receptor, EBC = early breast cancer, MBC = metastatic breast cancer, OS = overall survival, PFS = progression-free survival, TRR = tumor response rate, pos = positive, neg = negative.
